# Chimpanzees Preferentially Select Sleeping Platform Construction Tree Species with Biomechanical Properties that Yield Stable, Firm, but Compliant Nests

**DOI:** 10.1371/journal.pone.0095361

**Published:** 2014-04-16

**Authors:** David R. Samson, Kevin D. Hunt

**Affiliations:** 1 Department of Anthropology, University of Nevada, Las Vegas, Las Vegas, Nevada, United States of America; 2 Department of Anthropology, Indiana University, Bloomington, Indiana, United States of America; Georgia State University, United States of America

## Abstract

The daily construction of a sleeping platform or “nest” is a universal behavior among large-bodied hominoids. Among chimpanzees, most populations consistently select particular tree species for nesting, yet the principles that guide species preferences are poorly understood. At Semliki, *Cynometra alexandri* constitutes only 9.6% of all trees in the gallery forest in which the study populations ranges, but it was selected for 73.6% of the 1,844 chimpanzee night beds we sampled. To determine whether physical properties influence nesting site selection, we measured the physical characteristics of seven common tree species at the Toro-Semliki Wildlife Reserve, Uganda. We determined stiffness and bending strength for a sample of 326 branches from the seven most commonly used tree species. We selected test-branches with diameters typically used for nest construction. We measured internode distance, calculated mean leaf surface area (cm^2^) and assigned a tree architecture category to each of the seven species. *C. alexandri* fell at the extreme of the sample for all four variables and shared a tree architecture with only one other of the most commonly selected species. *C. alexandri* was the stiffest and had the greatest bending strength; it had the smallest internode distance and the smallest leaf surface area. *C. alexandri* and the second most commonly selected species, *Cola gigantea,* share a ‘Model of Koriba’ tree architecture. We conclude that chimpanzees are aware of the structural properties of *C. alexandri* branches and choose it because its properties afford chimpanzees sleeping platforms that are firm, stable and resilient.

## Introduction

Juvenile and adult apes devote considerable time and energy to the construction of a new sleeping platform or “nest” at the end of their daily active period [1]. Current data suggests that infants and young juveniles (i.e. nursing young) acquire skills over years both through observation of their mother and practice [1]–[3] and begin making their own nests after weaning. The universality of sleeping platform construction among the great apes suggests the behavior is genetically predisposed, though observation and learning are known to be critical [3]. Nest-builders must select an appropriate site, climb to the site and manipulate a large volume of foliage while maintaining balance. They bend and break stiff, strong stems [1], [2] as they incorporate foliage into the nest structure, pulling nest material inwards and interweaving it into a thick, springy ‘mattress,’ often bending branches in two. Ape nest mattresses are functionally concave in that either the edges are elevated over the mattress surface or the edges are less compliant than the center [4], causing the nest to assume a concave surface under pressure.

The function of sleeping platforms seems straightforward: a compliant yet constraining structure reduces stress on tissues and the functional concavity of the nests obviates the need to adjust posture during sleep to prevent falls. This *sleep quality hypothesis* holds that apes construct sleeping platforms to allow uninterrupted sleep and to promote longer individual sleep stages, resulting in a higher sleep quality [3], [5]–[9]. Additionally, captive orangutans exhibit higher quality sleep with less gross-motor movements and greater overall sleep times when using complex sleeping platforms [10]. The evidence for the sleep quality hypothesis seems compelling, yet observations of patterns of nest site selection, nest heights and the physical characteristics of the most-preferred tree species are taken by some researchers as supporting a different function for nesting (although these hypotheses need not be mutually exclusive), including *predation avoidance* [11], [12], *postural stability* (preventing falls assumed to be more likely because apes have great body mass relative to local supporting branch diameter [9], [13]), *thermoregulation* [7], [8], [14], and *pathogen avoidance*–either because the nest serves as a physical barrier to insect vectors such as mosquitoes [7], [8], [15] or because some chemical property of the species selected for nesting discourages mosquitoes [8], [16]. In accord with *predation avoidance,* chimpanzees appear to select sites both as far from the main stem or trunk as possible and with an escape route to neighboring trees due to canopy connectivity [11], [17]. In accord with the *sleep quality hypothesis*, tree species with smaller leaves and/or denser leaf distribution are argued to be selected to reduce stress on pressure points thus affording ‘comfort’ [4] and potentially effect thermoregulation [18]. Tree morphology may influence site selection because particular branching patterns such as the inverted tripod form is most easily manipulated into a bowl-shaped nest; chimpanzee preference at Bwindi for *Drypetes gerrardii* is hypothesized to be related to its “lollipop” crown shape and tripod morphology [19]. Furthermore, tree morphologies may be reinforced through time by way of long term re-use of trees, which shapes local branch ‘morphologies’ of potential sleep sites [20].

To the extent that tree species vary in morphology, physical characteristics such as stiffness and habitat preference, functional imperatives are expected to be expressed in the selection of particular tree species as sleeping sites. If the sleep quality hypothesis is correct, chimpanzees should prefer species that have smaller leaves, denser canopies and tripod-shaped branchings, compared to other available species. It follows from the thermoregulation hypothesis that species with dense, leafier canopies would be preferred. The predator-avoidance hypothesis entails a preference for tree species that are tall, are characterized by broad canopies and are distributed near waterways or gorges because slopes increase the functional height of nests. The antivector hypothesis suggests that species with certain volatile compounds in bark, sap or leaves will be preferentially selected.

Branch diameters of supporting structures have been shown to be an essential variable for understanding sleep site selection and canopy movement in orangutans, as local diameter is negatively related to compliance and positively related to length of the trunk [21]. Furthermore, is has been suggested that orangutans choose nest sites that afford stem diameters that yield optimal nest characteristics and that they manipulate nest materials in specific ways that demonstrate technical knowledge concerning structural properties of stems. Orangutan sleeping platforms have a high proportion of “greenstick fractures,” which are purported to impart a stronger and more resilient quality to supporting branches, suggesting that orangutans purposely break branches in this fashion during nest construction [22]. Semliki chimpanzee tree-species preference may indicate that chimpanzees display technical knowledge of the physical properties of raw materials similar to that of orangutans and that it leads them to choose particular species for nesting. Eastern chimpanzees (*Pan troglodytes schweinfurthii*) at three relatively dry sites (Ishaha [23], Budongo [24] and Toro-Semliki [25]) in western Uganda strongly prefer the species *Cynometra alexandri* (C.H.Wright) as raw material for sleeping platform construction. *C. alexandri* is common in dry or riverine forests throughout Central and East Africa [26] where the wood is often known locally as ‘ironwood’ or ‘muhindi’ in recognition of its dense, durable and resilient qualities; it is useful as a construction material [27]. Here we compare the structural qualities of seven common species of trees at Semliki to determine whether chimpanzees purposely select tree species that possess mechanical properties that determine superior nesting qualities.

A body mass of 30 kg or more means that chimpanzees require strong materials to contain their center of mass during sleep phases with a period of physical paralysis [9]. Furthermore, it is expected that materials with great stiffness would be preferred for construction of a springy mattress that can bear weight without over-compressing thus allowing contact with inflexible and uncomfortable large-diameter supports. If the physical properties of trees influence nesting site selection, we predict that the trees most commonly selected by chimpanzees will have significantly greater strength (capacity to bear stress before catastrophic failure), a higher elastic modulus or stiffness, significantly smaller internode distances (correlated with stiffness), tree architecture characterized by a high proportion of inverted tripod branchings and/or small leaf surface areas.

## Methods

### Ethics Statement

Authorization to conduct research inside Uganda was granted by the Government of Uganda. Permission to carry out research at the Toro-Semliki Wildlife Reserve was granted and approved through permits from the Uganda Wildlife Authority (UWA) and the National Research Council.

### Study Area

The Toro-Semliki Wildlife Reserve (TSWR) occupies 548 km^2^ in the Great Rift Valley from the Semliki River in the west to the top of the escarpment in the east, and from the foothills of the Ruwenzori Mountains in the south to the shore of Lake Albert in the north, northwest of Fort Portal, Uganda (0°50′ to 1°05′N, 30°20′ to 30°35′ E). The Wasa River runs from south to north through the center of the reserve, emptying into Lake Albert in the north. The reserve is crosscut by small, shallow, often seasonal water-courses that support narrow gallery forests ranging in width from 50–250 m [25]. The chimpanzee study community occupies the Mugiri River valley and eastwards up the escarpment slope to include valleys and seasonal tributaries to the Mugiri and the open woodland, bushland and grassland between the water-course; the community home range is the largest known of any site [28] and has been calculated to be 96 km^2^ (minimum convex polygon). The biome is predominantly dry *Combretum* and *Borassus* palm grassland with only 7.25% forest cover [25]. The habitat is hot and dry, with a daily maximum averaging 34°C and an average rainfall of 1389 mm (1996–2012), though earlier rainfall records vary between 700 and 1300 mm [26], [29]. The Mugiri community may have 150 members; 29 males have been identified, but few females are known as individuals; however, typical chimpanzee demography yields 60 females and 60 immatures [30].

### Sleeping Platform Tree Species

Phenology data on tree species distribution throughout the habitat was used from previous work [25]. Chimpanzee sleeping platforms were noted during the course of regular behavioral observation. Typical variables were recorded for each nest, including the species in which the nest was found. N = 1844 nests were included in the sample.

### Tree Architecture Classification

We followed van Wyk and colleagues [31] in assigning tree species used for nesting to one of 23 architectural model categories. Tree architecture is classified according to the developmental sequence of axes, or shoots [31]. Six tree architecture models were used for nest construction at Semliki (with associated prevalence throughout the home range): *Model of Attim* (9.9%), *Model of Fagerlind* (5.3%), *Model of Koriba* (58.2%), *Model of Leeuwenberg* (10.4%), *Model of Tomlinson* (4.4%), *and Model of Troll* (0.4%). We used *X^2^* analysis to assess chimpanzee preference for architectural models, relative to prevalence within the home range.

### Material Properties Measurement

Our methods closely followed Stewart and colleagues [4] by determining the biomechanical propeties of stems (a primary plant axis that develops buds and shoots instead of roots). Three-hundred and twenty six stems were used from the same multiple species generated in a sample of 65 nests from May–June 2008 and August 2010–January 2011; samples closely conformed to the length and diameter of “frame support branches” in nests (FSB diameter [N = 60, 4.13 cm ±1.11], see 9); FSB’s are branches that have been bent double and interwoven in the initial stages of nest construction and are used as primary weight bearing supports. The force in kg required to bend stems to 45°, 90° and to the point of structural failure were measured with a spring balance. We used the point of attachment of the spring balance and the distance from the anchor point to the break point to calculate torque, equal to the force applied multiplied by the distance between an object’s axis of rotation and the point where the force is applied [4]. A relative break force (RBF) value was calculated using a standardized measure of the force necessary to break any branch at a distance of 1 m. The RBF values were used to assess bending strength across tree species (One-way ANOVAs). *Beilschmiedia ugandensis* was an ideal control given its rare selection as a sleeping site and ubiquity throughout the home range and was featured in a LSD post hoc analysis of the RBF values compared among preferred species.

We measured internode lengths (N = 2574) along the stems of selected tree species often selected for sleeping sites to compare internode distances among genera (One-way ANOVA), using *Beilschmiedia ugandensis* as a control. We calculated the surface area of leaves (N* = *428) from selected tree species by tracing leaf outlines over 1 cm^2^ paper (then summing the number of squares within the outline) and compared species using a one-way ANOVA, again with *B. ugandensis* used as a control.

All statistical tests were 2-tailed, set at an alpha = 0.05 significance level.

## Results

**Figure 1:**
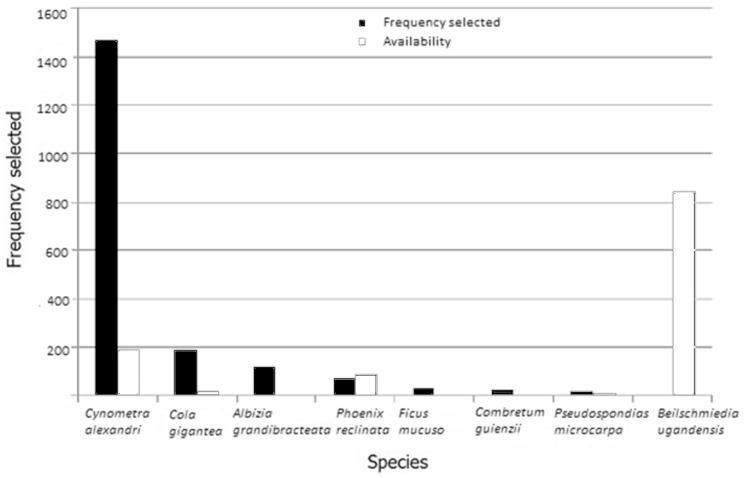
Top seven tree species selected by chimpanzees for sleeping platform construction at TSWR relative to species availability, included the control species *Beilschmiedia ugandensis* selected as a sleeping site <1% (adapted from [25]).

Of 1,844 nests sampled, *Cynometra alexandri* was selected for 73.6% of the nests, even though it represents only 9.6% of all trees on our 20 habitat-sampling transects (Fig. 1, [25]); this value is the second strongest preference for sleeping tree species recorded to date among all chimpanzee sites (Table 1). The second most commonly selected tree species at Semliki was *Cola gigantea,* constituting 9.2% of all recorded nests, but representing only 0.9% tree species in the habitat. The most common species sampled on our transects was *Beilschmiedia ugandensis,* representing 42.3% of all tree species. This most-common species was selected for nesting only 16 times, making up only 0.8% of all nests. Other species selected for nesting were *Albizia grandibracteata, Combretum molle, Ficus mucuso, Phoenix reclinata* and *Pseudospondias microcarpa.*

**Table 1:** Previously recorded chimpanzee sleeping tree species preference by field site.

Site	Tree species	%	Reference
*West Africa*			
Cantanhez, Guinea- Bissau	*Elaeis guineensis*	92.0	[34](11) et al. 2008
German-Fort, Gashaka- Gumti, Nigeria	*Khaya seneganensis* (dry season), *Craibia atlantica* (wet season)		[35]
Yealé, Nimba, Ivory Coast	*Chidlowia sanguinea*		[36]
Assirik, Senegal	*Spondias mombin, Adansonia digitata*		[13]
*Central Africa*			
Goualougo Triangle, Nouabalé-Ndoki,CongoSanz et al. 2007Ham 199	*Greenwayodendron suaveolens*	9.0	[37]
Ishasha, DRC	*Cynometra alexandri*		[23], [38]
Tishibati, Kahuzi-Biega, DRC	*Syzygium parvifolium*	21.8	[39]
Rio Muni, Equatorial Guinea	*Spondias mombin, Adansonia digiata*		[13]
Pongara, Gabon	*Coula edulis*	22.8	[40]
Seringbara Nimba, Guinea	*Amanoa bracteosa* (two study periods), *Childlowia sanguinea*(one study period)	11.910.7	[14], [36]
Wawba, DRC	*Leonardoxa romii*	26.4	[41]
Yalosidi, DRC	*Leonardoxa romii*	34.5	[42]
*East Africa*			
Gombe, TanzaniaGoodall 1962Hernandez-Aguilar 201	*Brachstegia bussei, Elaes grineensis*		[1]
Issa, Tanzania	*Brachystegia sp.*	36.1	[43]
Kasakati, Tanzania	*Cynometra sp.*		[44]
Lwazi, Tanzania	*Trichilia dregeana, Pseudospondia microcarpa, Dichapetalum stuhlmannii*		[45]
Ntakata/Kakungu, Tanzania	*Brachystegia bussei*	23.4	[46]
Ugalla, Tanzania	*Monopetalanthus richardsiae*	39.5	[47]
Kwitanga, Tanzania	*Brachstegia bussei*		[48]
Budongo, Uganda	*Cynometra alexandri*		[24]
Bwindi, Uganda	*Drypetes gerrardii*	21.0	[19]
Kalinzu, Uganda	*Uvariopsis congensis*	41.1	[48]
Kibale Ngogo, Uganda	*Uvariopsis congensis*	39.0	[49]
Semliki, Uganda	*Cynometra alexandri*	73.6	Current study, [25], [28]

Field sites recording up to three preferred species, without statistical supporting data, were included; citations where greater than three species were recorded as “preferred” were not included. Percentages were included when presented.

In a one-way ANOVA test *C. alexandri* was the stiffest and most stress resistant of the eight species we tested, with the highest relative break force (RBF) greater than the other seven species in three of four diameter categories (see Table 2 for RBF distributions and Table 3 for post hoc analysis), category 1, <3 cm (F [6, 199]) = 11.49, *P*<0.001), category 2, 3–4.9 cm (F [6, 155] = 6.41, *P*<0.001), category 3, 5–6.9 cm (F [6, 50] = 10.80, *P*<0.001). Size category 4, 7–9 cm, (F [4], [16] = 0.84, *P* = 0.524) did not differ significantly from other species; however, our sample contained very few branches in this diameter category (Table 2).

**Table 2:** Relative break force values (scores 1–4) of the preferred species used to construct SPs (including the control *Beilschmiedia ugandensis*); total N = 441 tested breaks and generated RBF values.

Diameter Score	Top seven genera RBF values	Mean and SD	Range	N total per species
1 (<3 cm)	*Cynometra*	213.7±116.9	34–721	78
	*Cola*	120.7±62.0	71–248	8
	*Pseudospondias*	107.9±67.54	22–312	67
	*Combretum*	107.5±55.4	37–192	7
	*Albizia*	100.1±70.3	26–331	33
	*Beilschmeidia*	100.0±62.9	33–220	9
	*Phoenix*	86.7±36.1	52–124	3
2 (3–5 cm)	*Cynometra*	531.8±284.4	120–1650	54
	*Pseudospondias*	408.5±33.2	103–1063	48
	*Beilschmeidia*	367.3±152.1	220–670	9
	*Combretum*	353.0	n/a	1
	*Phoenix*	270.8±224.4	55–568	4
	*Cola*	253.4±147.1	82–607	27
	*Albizia*	240.9±129.0	56–445	20
3 (5–7 cm)	*Cynometra*	1493.9±473.6	856–2200	13
	*Pseudospondias*	1019.9±420.4	469–1550	8
	*Beilschmeidia*	838.5±166.3	700–1068	4
	*Combretum*	596.0±567.9	196–1246	3
	*Phoenix*	589.8±64.9	499–653	4
	*Cola*	520.5±217.9	259–1104	14
	*Albizia*	504.0±293.0	215–1052	9
4 (7–9 cm)	*Cynometra*	1630.3±460.88	1301–2306	4
	*Beilschmeidia*	1364.5±459.3	791–2200	7
	*Cola*	1340.0±721.2	830–1850	2
	*Combretum*	1118.0±538.4	530–1800	4
	*Albizia*	766.0	n/a	1

**Table 3:** Least Significant Difference (LSD) post hoc analysis comparing the control species *Beilschmeidia ugandensis* to preferred sleeping tree species (score 4 was omitted due to multiple groups having fewer than two cases).

Diameter Score	Genus	Mean difference	Standard error	Significance
1 (<3 cm)				
*B. ugandensis*	*Cynometra*	−113.7	35.9	*P<*0.01*
	*Cola*	−20.7	50.0	*P = *0.68
	*Pseudospondias*	−7.9	36.2	*P = *0.83
	*Combretum*	−7.5	52.2	*P = *0.87
	*Albizia*	−0.6	38.2	*P = *0.99
	*Phoenix*	13.3	65.4	*P = *0.84
2 (3–5 cm)				
*B. ugandensis*	*Cynometra*	−164.6	81.7	*P = *0.05*
	*Albizia*	−126.4	92.4	*P = *0.17
	*Pseudospondias*	−41.3	82.4	*P = *0.62
	*Combretum*	−7.5	52.2	*P = *0.87
	*Phoenix*	86.5	131.9	*P = *0.47
	*Cola*	113.9	87.1	*P = *0.19
3 (5–7 cm)				
*B. ugandensis*	*Cynometra*	−655.4	180.1	*P<*0.01*
	*Pseudospondias*	−181.4	195.6	*P = *0.36
	*Phoenix*	248.8	220.6	*P = *0.26
	*Cola*	318.0	178.4	*P = *0.08
	*Albizia*	334.5	191.1	*P = *0.09

For each diameter score, *Cynometra* is uniquely positioned as significant with the greatest RBF value relative to the control.

In a one-way ANOVA, *C. alexandri* had significantly smaller internode distances compared to seven comparison species (N = 150, 1.47±1.3 cm, range: 0–9; F [6, 1219] = 13.20, *P*<0.001). In a one-way ANOVA *C. alexandri* had significantly smaller leaf surface areas than other species (N = 83, 3.77±2.9 cm, range: 0.25–18; F [4, 240] = 140.31, *P*<0.001). The second most commonly selected tree species, *C. gigantea,* had the largest leaf surface area (N* = *33, 208.14±112.4 cm, range: 17.5–426.25).

Of the seven architectural models characterizing trees in the TSWR, four were commonly used by chimpanzees: *Model of Troll* (characteristic of *Albizia;* N = 120); *Model of Leewenberg* (both *Combretum* [N = 24] and *Ficus* [N* = *30]); *Model of Tomlinson* (*Phoenix* [N* = *70]); *Model of Koriba* (*Cynometra* [N* = *1430], *Cola* [N = 189], and *Pseudospondias* [N* = *19]). *Koriba* was the most preferred tree architecture, selected for 90.0% of all trees, significantly greater than expected by chance (*X^2^* = 1013.9, df = 6, P<0.001; see Table 4).

**Table 4:** Tree architecture models preferentially chosen as sleep sites by TSWR chimpanzees.

Architectural model	Observed as sleep site	Expected # to be observed as sleep site	Expected % based off of species distribution
Koriba	1660	1073	58.17
Troll	120	52	2.83
Leeuwenberg	54	237	12.83
Fagerlind	8	100	5.31
Attim	2	183	9.94
Tomlinson	70	126	6.81

## Discussion

Our results strongly suggest that just as do orangutans [22], chimpanzees are selective of tree species when considering where to sleep. These data further suggest that chimpanzees select species of trees that possess physical properties that result in nests that are sturdy and resilient, optimizing comfort and reducing the risk of falls. The preferred species at Semliki, *C. alexandri* has the greatest relative break force and the smallest internode distance of the species tested; both promote stiffness. Because sleeping platforms utilize the *basketweave* (a variation of the *plain weave* [the most fundamental type of weave with a simple crisscross pattern] in which two are more stems can be bundled and then woven as one) [32], smaller internode distances may also introduce a greater number of interlocking points, yielding greater structurally integrity and resilience. Future research directions should investigate the mechanisms by which chimpanzees consider the physical properties of stems during platform construction and whether weaving patterns produce biomechanically sturdier sleeping platforms.

*C. alexandri* had the smallest leaves with the greatest density (i.e., internode distance) of all nesting tree-species, which has been suggested as affording the greatest comfort [18] by reducing exposure to branches protruding from the nest structure and increasing friction among interlocking of stems, creating a tighter, more securely woven structure. By providing thicker foliage, it may also increase insulation and thus offer thermoregulatory advantages. Finally, *C. alexandri* may also have insect-repellent properties [16].

Among the preferred tree species at Semliki (Fig. 1) was *Phoenix reclinata,* a palm with physical characteristics dramatically different from the most-preferred species. *P. reclinata* has pinnate, or feather-shaped leaves 3–5 cm in breadth and 40 cm in length. The leaves are stiff and the base of the leaf stem has thorns that incorporate paralytic secondary compounds [33]. Use of palms for nest manufacture is common and highly preferred at some sites (refs. in Table 1); palms are the preferred species in southern Guinea-Bissau [34]. We consider the selection of this species to be at odds with most hypotheses for nest construction: it rarely interdigitates with other canopies; it has no lateral branches; it is a short tree (relative to other nesting species); leaves are large; and interweaving is difficult. Sousa and colleagues [34] hypothesized that oil palm are selected for their antipredator qualities including increased line of sight (given the spatial location of oil palm was significantly situated at forest edge), increased communication possibilities with conspecifics and improved access to resources. We additionally hypothesize that the paralytic secondary compounds associated with thorny leaf stems and protruding, jagged trunk morphology attribute the antipredition characteristics associated with this species. Furthermore, it may be that oil palm is preferred because if affords maximal stiffness in substrate, given it is characterized by having no lateral branches and the weight bearing structure is the trunk itself.

**Figure 2:**
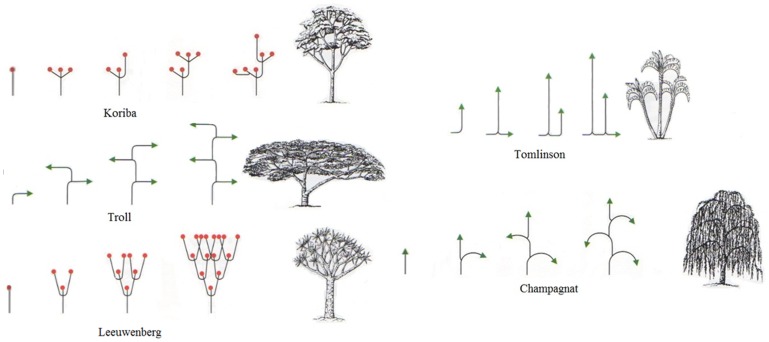
*Model of Koriba* (above left corner) is the specialized tree architecture preferred by Semliki chimpanzee for use as a sleeping site. Notice the shoot development which produces a node dense, leafy and sturdy substrate with multiple frame supporting branches (FSBs) with which to construct a sleeping platform. In contrast, the shoot development which produces lateral drooping (inferred low levels of stiffness) and long internode distances illustrated by *Model of Champagnat* (below right corner), provides a poor sleeping substrate and is never selected by Semliki chimpanzees. Models Koriba, Troll and Leeuwenberg comprise 96.2% of all selected species and share the “lollipop” end static tree shape (adapted from [31]).

Our results are compatible with the hypothesis that chimpanzees select trees that are configured similar to the lollipop morphology of *Drypetes gerrardii* [19]. The model of Koriba *C. alexandri* tree architecture affords chimpanzees inverted-tripod nest frames, which are hypothetically the easiest to transform into the functionally concave form to maximize comfort and safety [22]. Other species selected less preferentially than *C. alexandri* likewise support this hypothesis: three of the four (96.2% of all selected species) tree architectures used by chimpanzees at Semliki possess inverted tripod branching patterns (Fig. 2). The tree architecture *Tomlinson* is a unique case, as it is an oil-palm tree with a markedly different shoot development.

## Conclusions

Our results suggest that the ideal sleeping platform tree species might well possess multiple advantages, perhaps possessing antipredator, antivector, thermoregulatory and comfort- maximizing qualities all at once. *C. alexandri* has all of these properties, suggesting that chimpanzees are keen observers of physical properties of trees, including stiffness, strength and leaf surface area, and that they select species that provide the widest range of advantages, including predator avoidance, postural stability, thermoregulation and pathogen avoidance.
