# Correction: Chimpanzees Preferentially Select Sleeping Platform Construction Tree Species with Biomechanical Properties that Yield Stable, Firm, but Compliant Nests

**DOI:** 10.1371/journal.pone.0104024

**Published:** 2014-07-24

**Authors:** The PLOS ONE Staff

There are errors in Table 1. The authors have provided a corrected Table 1 here.

**Table 1:** Previously recorded chimpanzee sleeping tree species preference by field site.

Site	Tree species	%	Reference
*West Africa*			
Cantanhez, Guinea- Bissau	*Elaeis guineensis*	92.0	(34)
German-Fort, Gashaka- Gumti, Nigeria	*Khaya seneganensis* (dry season), *Craibia atlantica* (wet season)		(35)
Yealeì, Nimba, Ivory Coast	*Chidlowia sanguinea*		(36)
Assirik, Senegal	*Spondias mombin, Adansonia digitata*		(13)
*Central Africa*			
Goualougo Triangle, Nouabaleì-Ndoki, Congo	*Greenwayodendron suaveolens*	9.0	(37)
Ishasha, DRC	*Cynometra alexandri*		(23, 38)
Tishibati, Kahuzi-Biega, DRC	*Syzygium parvifolium*	21.8	(39)
Rio Muni, Equatorial Guinea	*Spondias mombin, Adansonia digiata*		(13)
Pongara, Gabon	*Coula edulis*	22.8	(40)
Seringbara Nimba, Guinea	*Amanoa bracteosa* (two study periods), *Childlowia sanguinea* (one study period)	11.9 10.7	(14, 36)
Wawba, DRC	*Leonardoxa romii*	26.4	(41)
Yalosidi, DRC	*Leonardoxa romii*	34.5	(42)
*East Africa*			
Gombe, Tanzania	*Brachstegia bussei, Elaes grineensis*		(1)
Issa, Tanzania	*Brachystegia sp.*	36.1	(43)
Kasakati, Tanzania	*Cynometra sp.*		(44)
Lwazi, Tanzania	*Trichilia dregeana , Pseudospondia microcarpa, Dichapetalum stuhlmannii*		(45)
Ntakata/ Kakungu, Tanzania	*Brachystegia bussei*	23.4	(46)
Ugalla , Tanzania	*Monopetalanthus richardsiae*	39.5	(47)
Kwitanga, Tanzania	*Brachstegia bussei*		(48)
Budongo, Uganda	*Cynometra alexandri*		(24)
Bwindi, Uganda	*Drypetes gerrardii*	21.0	(19)
Kalinzu, Uganda	*Uvariopsis congensis*	41.1	(48)
Kibale Ngogo, Uganda	*Uvariopsis congensis*	39.0	(49)
Semliki, Uganda	*Cynometra alexandri*	73.6	Current study, (25, 28)

Field sites recording up to three preferred species, without statistical supporting data, were included; citations where greater than three species were recorded as “preferred” were not included. Percentages were included when presented.
